# A Review on Climate Change Redefining Tetrodotoxin Accumulation and Ecological Dynamics in Pufferfishes

**DOI:** 10.3390/md24090300

**Published:** 2026-08-30

**Authors:** Govind Choudhary, Adeeba Hamdani, Hideaki Unno, Masanari Kimura, Balu Alagar Venmathi Maran

**Affiliations:** 1 Graduate School of Integrated Science and Technology, Nagasaki University, 1-14 Bunkyomachi, Nagasaki 852-8521, Japan; govind.1999thechoudhary@gmail.com (G.C.); unno@nagasaki-u.ac.jp (H.U.); masanari@nagasaki-u.ac.jp (M.K.); 2 Department of Aquaculture, College of Fisheries, Ratnagiri 415629, Maharashtra, India; adibahamdani123@gmail.com; 3 Organization for Marine Science and Technology, Nagasaki University, 1-14 Bunkyomachi, Nagasaki 852-8521, Japan

**Keywords:** tetrodotoxin, *Takifugu*, ocean warming, hypoxia, ocean acidification, microbial ecology, trophic transfer, hybridization, biogeographic range shift, LC-MS/MS

## Abstract

Pufferfishes (Tetraodontidae) accumulate tetrodotoxin, a potent neurotoxin, primarily through microbial and trophic pathways rather than endogenous biosynthesis, linking their toxicity directly to marine microbial ecology and food-web structure. Anthropogenic climate change, ocean warming, deoxygenation, and acidification are reshaping the environmental conditions that govern TTX production, transfer, and accumulation. This review synthesizes evidence from 62 studies (28 with quantitative data) identified through a PRISMA-ScR-compliant search. We organize findings around three mechanistic pathways: (1) warming-associated proliferation of TTX-producing bacteria and consequent shifts in environmental TTX availability, (2) climate-driven range shifts and hybridization that generate novel and unpredictable toxicity phenotypes, and (3) physiological responses of pufferfishes to warming, hypoxia, and acidification that may alter toxin allocation. We identify priority research needs, long-term monitoring, multi-stressor experiments, and standardized LC-MS/MS-based surveillance and outline an integrated framework for anticipating climate-driven shifts in pufferfish toxicity.

## 1. Introduction

Tetraodontid pufferfishes are widely recognized for their specialized anti-predator morphology and the high-affinity sequestration of tetrodotoxin (TTX) within specific organ systems [1]. Acting through the selective occlusion of voltage-gated sodium channels, TTX provides an effective chemical defence and mediates conspecific reproductive signalling in certain species [2]. The origin of TTX in pufferfishes is exogenous, acquired via dietary ingestion and microbial biosynthesis, thereby linking organismal toxicity directly to ambient microbial ecology and benthic food-web interactions [3,4]. Furthermore, host–parasite interfaces represent an active route of toxin exchange: the ectoparasitic copepod *Caligus fugu* (=*Pseudocaligus fugu*) infesting *Takifugu pardalis* exhibits significant TTX bioaccumulation and harbours culturable TTX-associated bacteria [5,6,7], demonstrating that parasitic interactions constitute an integral yet undercharacterized component of marine TTX trophic dynamics. The sensitivity of TTX to climate perturbation stems directly from its non-autonomous biosynthetic pathway, which integrates microbial physiology with multi-trophic transfer cascades. Primary production by bacterial taxa including *Vibrio*, *Aeromonas*, and *Pseudomonas* [4], coupled with downstream trophic consumption networks [8], is profoundly governed by prevailing temperature, oxygenation, and pH gradients. As a result, marine climate drivers like ocean warming, hypoxia, and acidification modulate TTX bioaccumulation through indirect, interconnected ecosystem pathways rather than direct physiological pressure on the fish host alone [1,3]. This climate coupling is exemplified by field observations of *T. pardalis*, where peak somatic and gonadal TTX concentrations align closely with seasonal sea surface temperature elevations [9], consistent with the hypothesis of thermally augmented bacterial proliferation and accelerated trophic uptake. Anthropogenic climate forcing is fundamentally restructuring the physicochemical template of marine ecosystems, with sea surface warming, deoxygenation, and ocean acidification driving widespread shifts in plankton composition, microbial assemblages, and biogeographic ranges [1,10,11]. For an ecologically vectored neurotoxin like TTX, these perturbations carry disproportionate consequences because microbial growth kinetics, secondary metabolite biosynthesis, and trophic network dynamics are intricately coupled to thermal and geochemical regimes [10,11]. Corroborating this climate vulnerability, accumulating evidence links elevated ocean temperatures to the enhanced proliferation of toxigenic bacterial consortia, seasonal surges in organ-specific TTX titers, and the emergent detection of TTX across previously non-toxic geographic regions and taxonomic clades [8,9,12]. Climate-induced poleward range shifts, predominantly driven by accelerating warm-water currents, are generating novel zones of sympatry among historically isolated tetraodontid species and facilitating introgressive hybridization [10,11]. Hybrid individuals frequently exhibit non-canonical toxin compartmentalization that diverges markedly from parental phenotypes—including unexpected TTX bioaccumulation in tissues conventionally deemed safe—thereby introducing significant anatomical toxicity unpredictability that challenges species-specific food-safety frameworks [13,14]. Concurrently, the compounding impacts of ocean warming, hypoxia, and acidification (multi-stressor regimes) are anticipated to modulate ontogeny, phenological reproductive timing, and internal toxin trafficking, though rigorous empirical evidence disentangling these synergistic interactions within TTX bioaccumulation dynamics remains exceedingly scarce [15]. In light of these nested ecological interactions, pufferfish toxicity is best characterized as a dynamic, context-dependent process governed by environmental drivers rather than a fixed evolutionary attribute. Here, we implement a rigorous scoping synthesis to evaluate how climate-induced ocean alterations affect TTX biogenesis, trophic transfer, and organismal accumulation across global marine systems. A central objective is to formally distinguish validated mechanisms [M] from correlative patterns and emerging hypotheses [H] (Section 2.4), thereby defining critical empirical and monitoring priorities required to forecast neurotoxin proliferation under future climate regimes.

## 2. Literature Search, Screening Methodology, and Evidence Classification

### 2.1. Definitions

For the purposes of this review, we define climate-linked TTX dynamics as any documented or hypothesized change in TTX production, environmental availability, trophic transfer, tissue accumulation, or human exposure risk that is associated with, or plausibly driven by, ocean warming, deoxygenation, acidification, or related habitat change (e.g., range shifts, altered stratification). This definition deliberately spans three evidence tiers (mechanism, association, and hypothesis; see Section 2.4) because the underlying literature is heterogeneous in design and directness of evidence.

### 2.2. Search Strategy

This review follows the Preferred Reporting Items for Systematic Reviews and Meta-Analyses extension for Scoping Reviews (PRISMA-ScR). Three bibliographic databases were searched for records published between January 2000 and December 2024. The core Boolean search string, adapted for each database’s syntax, was:

(“Takifugu” OR “pufferfish*” OR “puffer fish” OR Tetraodontidae) AND (“tetrodotoxin” OR “TTX”) AND (“climate change” OR “ocean warming” OR “sea surface temperature” OR “hypoxia” OR “ocean acidification” OR “deoxygenation” OR “range shift” OR “hybridization”)

Region-specific supplementary searches combined the above core string with the terms “Japan,” “Korea,” “China,” “Mediterranean,” “Southeast Asia,” “Thailand,” “Philippines,” “Indonesia,” “Oceania,” and “Indo-Pacific” to ensure broad geographic coverage. Truncation (*) was applied where supported. Database-specific yields were: Scopus, *n* = 176, PubMed, *n* = 134, Google Scholar (first 200 results per query, screened manually), *n* = 202, combined total: 512 records*.

### 2.3. Screening Protocol and Inter-Rater Reliability

All records were imported into reference-management software (Zotero, version 6.0.27, Corporation for Digital Scholarship) and de-duplicated, leaving 381 unique records. Titles and abstracts were screened independently against the following criteria.

**Inclusion criteria**: (i) Peer-reviewed primary research, review, or authoritative grey-literature report, (ii) original data on TTX occurrence or concentration in marine pufferfishes, or on pufferfish ecology/physiology in relation to an environmental variable (temperature, dissolved oxygen, pH, salinity, or habitat), (iii) published in English, 2000–2024.

**Exclusion criteria**: (i) Non-marine species without a marine pufferfish component, (ii) non-TTX toxins with no TTX component, (iii) conference abstracts lacking full methods, (iv) opinion pieces or commentaries without primary data.

**Figure 1:**
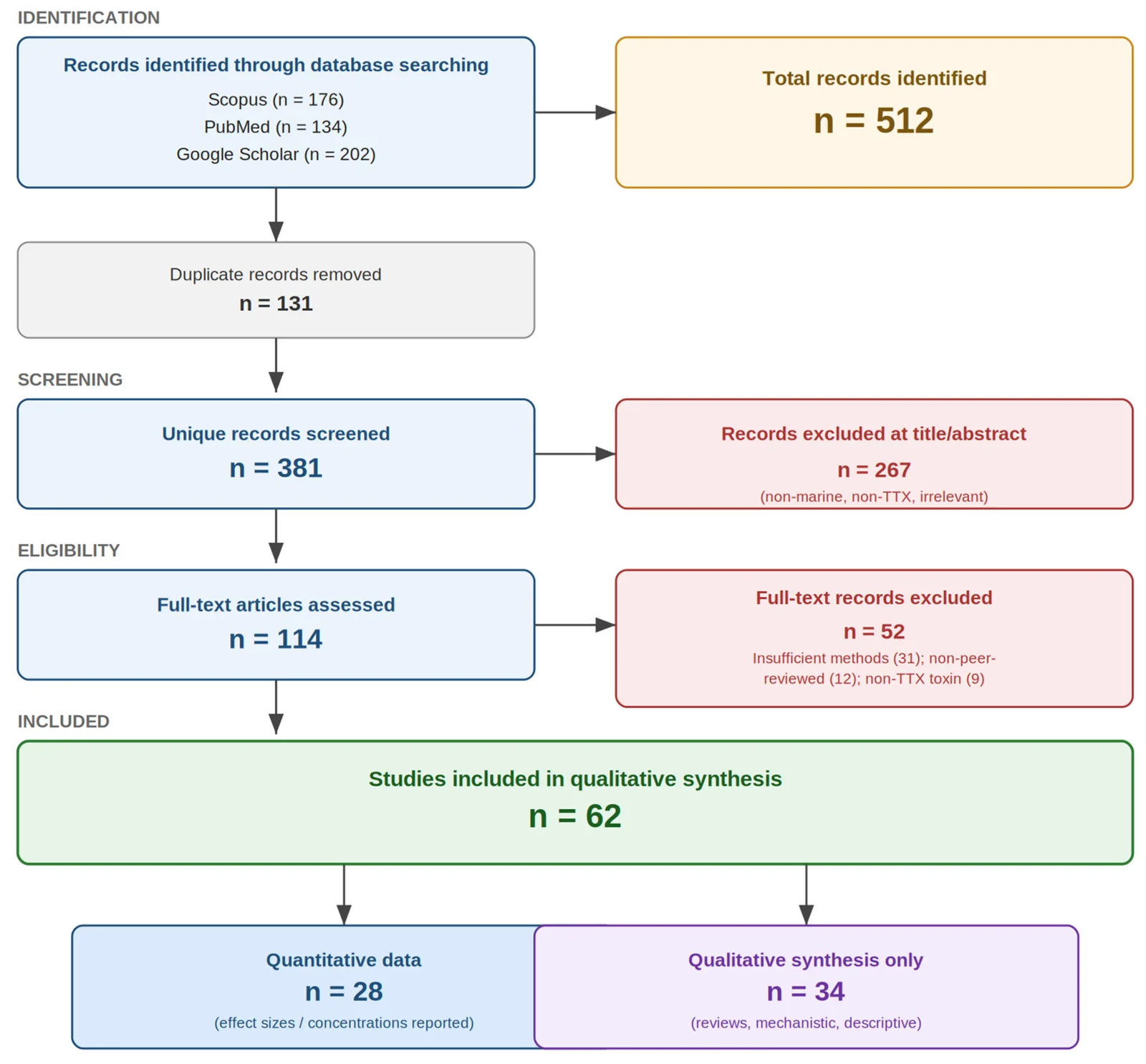
PRISMA-ScR flow diagram of the literature search and screening process [16]. Records identified through database searching (Scopus *n* = 176, PubMed *n* = 134, Google Scholar *n* = 202, total *n* = 512) were de-duplicated (*n* = 381 unique), screened at title/abstract stage (*n* = 267 excluded), assessed at full-text stage (*n* = 114), and a final set of 62 studies was retained for qualitative synthesis, of which 28 provided quantitative data suitable for detailed comparative discussion.

Title/abstract screening excluded 267 records as clearly irrelevant. Full texts of the remaining 114 records were independently assessed by both reviewers; 52 records were excluded at the full-text stage (31 lacking methodological detail, 12 non-peer-reviewed, 9 addressing non-TTX toxins). Agreement at the title/abstract stage was substantial (Cohen’s κ = 0.79); two unresolved disagreements were adjudicated. This process yielded 62 studies retained for qualitative synthesis (Figure 1). Of these, 28 studies contained sufficiently detailed quantitative data to support comparative discussion of climate-linked TTX variability; the remaining 34 informed the qualitative and mechanistic synthesis. Additional methodological and contextual references (e.g., PRISMA guidelines and supporting literature on bacterial toxins) were cited where relevant, resulting in a total bibliography of 66 references.

### 2.4. Statistical Analysis and Quantitative Synthesis

We employed a two-tiered quantitative approach: (1) descriptive statistics characterizing publication trends, study designs, and climate variables addressed; and (2) harvest plot analysis synthesizing directional outcomes of climate-TTX associations across the 28 quantitative studies. Data extraction included bibliographic information, study design, geographic scope, climate variables examined, TTX measurement methods, and quantitative outcomes. Proportions and frequencies were calculated in Excel. Direction consistency was classified as consistent (>70% agreement), mixed (40–70%), or conflicting (<40%).

#### 2.4.1. Publication Trends and Research Characteristics

Among the 62 included studies, research effort has increased markedly since 2015, is concentrated in East Asia (particularly Japan), and is dominated by field observational designs over laboratory experiments (Table 1).

#### 2.4.2. Harvest Plot Analysis: Outcome Synthesis

Of 28 quantitative studies, harvest plot analysis stratified outcomes by climate driver (Table 2). Temperature (24 studies): 14 (58%) reported increased TTX with warming, 7 (29%) mixed findings, 3 (13%) decreased TTX, indicating mixed direction. Median effect: +8.3% per 1 °C (range −2.4% to +24%). Hypoxia (11 studies): 8 (73%) reported increased TTX, 2 (18%) mixed, 1 (9%) decreased, consistent direction. Median effect: +15.2% per 1 mg/L O2 decline. Ocean acidification (5 studies) and salinity (7 studies) showed conflicting findings (Table 2). Multi-stressor studies (*n* = 6) suggested synergistic amplification of TTX under combined warming + hypoxia.

#### 2.4.3. Effect of Heterogeneity and Study Context

Experimental studies (*n* = 5) reported larger warming effects (median +12.1% per °C) versus field studies (*n* = 19; +6.8% per °C). Liver TTX increased more robustly (+9.1%) than gonadal/muscle tissue (+4.2%). While short-term experiments (*n* = 7) yielded highly variable results, long-term studies lasting over six months (*n* = 16) revealed much more consistent trends.

Hypoxia emerged as the most consistent climate driver of TTX elevation, while temperature effects remain heterogeneous across populations and tissues. Ocean acidification is understudied (13% of studies), and multi-stressor interactions warrant further investigation. These patterns define research priorities for climate-TTX ecology.

### 2.5. Evidence Classification Framework

We classify each major claim into one of three tiers because many statements in the climate–TTX literature mix experimental, observational, and theoretical evidence:

**[M] Confirmed mechanism**: Supported by controlled experimental studies directly testing a causal pathway (e.g., bacterial toxin-production kinetics across a temperature gradient);

**[SA] Strong association**: Supported by consistent field-observational data showing a correlation between an environmental variable and a TTX-related outcome, without experimental manipulation; alternative explanations are acknowledged;

**[H] Hypothesis**: A plausible, mechanistically motivated prediction not yet directly tested, explicitly flagged as a research priority.

The pronounced diversity of investigative frameworks (laboratory toxicology, ecological surveys, functional genomics) and incongruent endpoints (MBA units, analytical LC-MS/MS titers, qPCR-derived expression ratios, mortality rates) precluded a standardized meta-analysis. Accordingly, we employed a qualitative scoping synthesis following PRISMA-ScR principles tailored for complex, multi-tiered studies, while systematically integrating reported empirical metrics (means, observational ranges, and relative percentages) to maintain quantitative fidelity throughout the review.

## 3. Pufferfish Biology, Genomic Context, and Global Distribution

### 3.1. Evolutionary Background and the Takifugu Species Complex

The genus *Takifugu* encompasses approximately 25 valid species that underwent rapid adaptive radiation in the Northwest Pacific during the Pliocene (~1.8–5.3 Ma)—a diversification tempo rivalling the iconic cichlid radiations of the African Great Lakes [17]. Despite clear morphological divergence, high-resolution phylogenomics and Bayesian clustering reveal exceptionally low pairwise nucleotide divergence across the clade, indicating a genetically near-continuous species complex with incomplete reproductive isolation [17]. This genetic cohesion directly impacts toxin dynamics by facilitating interspecific hybridization and the introgression of key genetic architectures governing TTX handling, such as loci encoding TTX-binding proteins and ABC transporters [18]. Furthermore, the *Takifugu* genome is renowned for its remarkable compaction (~365–400 Mb, encoding ~19,000–22,000 genes) and high vertebrate gene density [19]. Two specialized genomic adaptations are particularly relevant to climate–TTX coupling: (1) atypical cardiospecific expression of erythropoietin (*epo*), conferring distinct hypoxic response dynamics compared to standard teleost renal pathways [20]; and (2) functional expansion of osmoregulatory gene families in euryhaline taxa (e.g., *T. obscurus*), conferring elevated resilience to climate-driven salinity fluctuations [21].

### 3.2. Global Distribution and Regional Toxicity Patterns

Pufferfishes (approximately 200 species and 27 genera) are distributed across tropical, subtropical, and temperate coastal, estuarine, and oceanic habitats globally [22]. Regional patterns of TTX occurrence and monitoring capacity are markedly uneven.

**Northwest Pacific (Japan, Korea, China).** This region harbours the greatest *Takifugu* species diversity and has the most developed TTX monitoring infrastructure. Japan has operated systematic pufferfish toxicity surveillance since the 1980s [20], and Korea maintains routine seasonal monitoring of commercial species including *T. pardalis* and *T. niphobles* [10,21]. China is one of the world’s largest producers of farmed pufferfishes (*T. rubripes*, *T. obscurus*, *T. fasciatus*), yet climate-linked TTX monitoring in aquaculture settings remains limited. Ji et al. [23] documented TTX in ovaries of farmed *T. rubripes* from Shandong Province (125 mouse units g^−1^ tissue in March), and Li et al. [24] isolated a novel TTX-producing bacterium (*Raoultella terrigena*) from wild *T. niphobles* collected in Hong Kong, findings consistent with environmental TTX availability throughout the region but underscoring the limited temporal and spatial scope of Chinese climate-focused studies.

**Mediterranean Sea**. The introduction and rapid spread of *Lagocephalus sceleratus* through the Suez Canal exemplifies a climate-accelerated Lessepsian migration with major sanitary and toxicological ramifications. First recorded in the Levantine basin in 2003 [13], the species rapidly extended its range westward, driven by thermal niche availability; species distribution modelling demonstrates that warming Mediterranean waters enhanced establishment dynamics and will facilitate prospective colonization toward the Adriatic Sea, Ligurian Sea, and southern coastline. Tissue TTX levels routinely surpass international regulatory limits, triggering critical clinical poisonings throughout the Eastern Mediterranean region [25].

**Southeast Asia**. Thailand, Vietnam, the Philippines, and Indonesia share long traditions of pufferfish consumption, yet systematic TTX monitoring programmes are absent or extremely limited. Multiple *Takifugu*, *Lagocephalus*, and *Arothron* species inhabit these coastal waters, often in regions undergoing rapid warming and coastal habitat degradation [26]. Poisoning incidents are documented but rarely enter the primary peer-reviewed literature in English, creating a critical evidence gap.

**Indo-Pacific and Oceania**. Tropical coral-associated pufferfishes (*Canthigaster* spp., *Arothron* spp.) occur throughout the Indo-Pacific, Oceania, and Great Barrier Reef systems. Stump et al. [17] documented warming-induced coral bleaching as a threat to coral-associated pufferfishes via destruction of refugia and feeding grounds; however, almost no climate-linked TTX data exist for these taxa outside the *Takifugu* complex.

**Indian Ocean and Arabian Gulf**. Pufferfishes (*Lagocephalus* spp., *Arothron* spp.) are present along East African and Arabian Gulf coastlines, and consumption-related poisonings are documented, but these regions remain essentially unstudied in terms of climate–TTX coupling, despite being among the most rapidly warming marine regions on Earth.

This geographic pattern reveals a stark imbalance: the regions with the best mechanistic evidence (Japan, Korea) represent only a fraction of global pufferfish diversity and human exposure risk. Tropical and subtropical regions, where climate warming rates are high and monitoring infrastructure is least developed, remain critical blind spots.

### 3.3. Why Pufferfishes Are Sentinel Species for Climate–TTX Research

**Figure 2:**
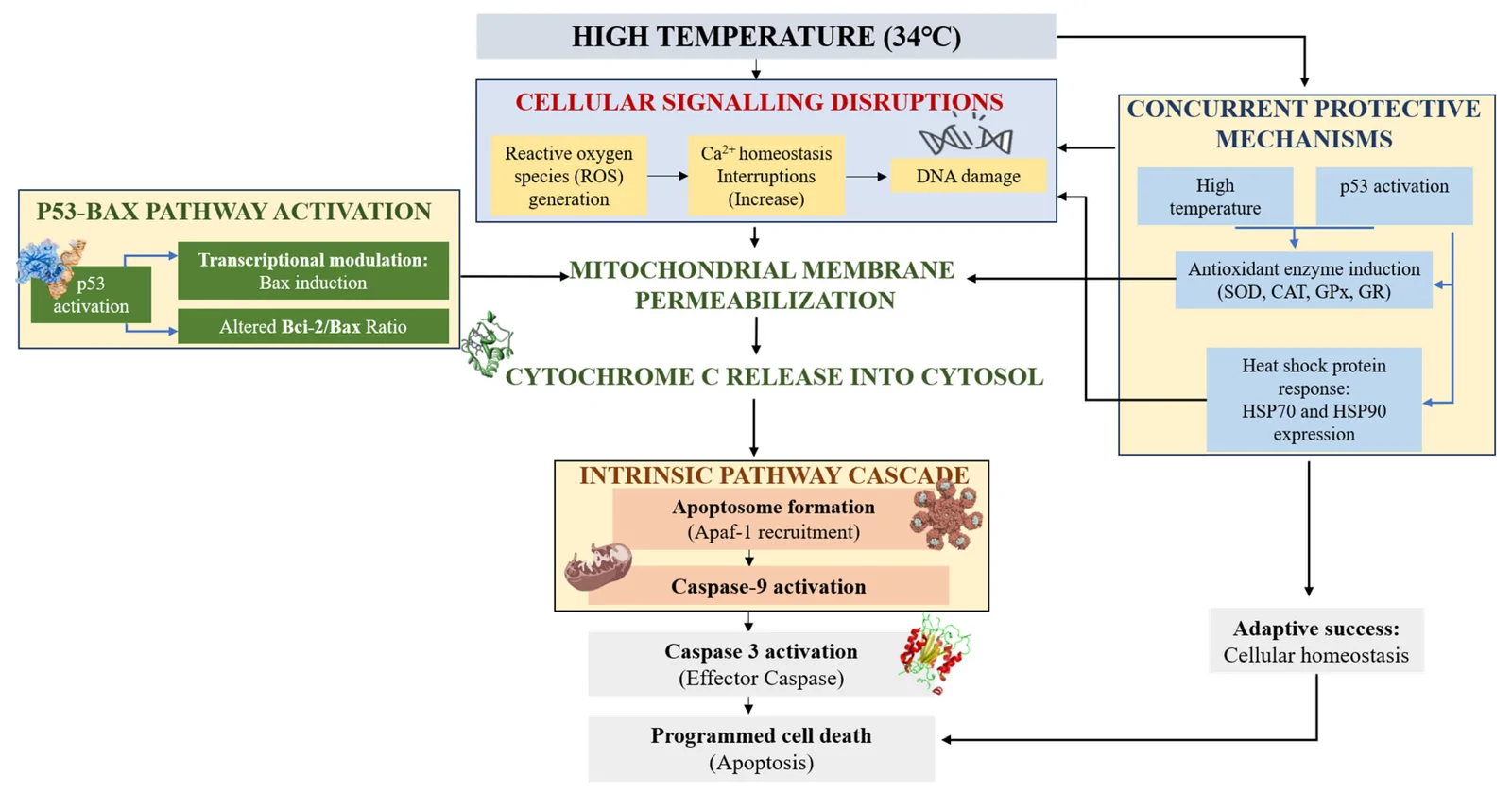
Thermal stress-induced apoptosis and protective responses in pufferfishes [29,31]. High temperatures (>30 °C for most *Takifugu*) induce reactive oxygen species (ROS) accumulation, calcium imbalance, and DNA strand breaks. Activation of the p53–BAX pathway initiates the intrinsic apoptotic cascade via caspase activation, leading to programmed cell death. Concurrent induction of antioxidant enzymes (SOD, CAT) and heat-shock proteins (HSP70, HSP90) provides protective buffering but carries energetic costs that may reduce growth and reproductive investment. These energetic trade-offs reduce the metabolic capacity available for TTX accumulation and translocation to reproductive tissues via ABC transporters, with direct implications for toxin allocation, maternal provisioning of eggs, and overall ecological toxin availability in warming coastal systems [18,20].

Three converging characteristics make *Takifugu* pufferfishes uniquely suited as biological sentinels for tracking climate-induced neurotoxin dynamics [8,12]. Primarily, the exogenous origin of TTX ensures that host toxin profiles directly integrate real-time trophic bioavailability and ambient microbial productivity. Under elevated thermal regimes, the metabolic investment required for antioxidant upregulation and heat shock response suppresses active toxin sequestration pathways [8,27,28]; heat-induced activation of the p53–BAX axis further limits toxin processing efficiency through significant cellular stress expenditures (Figure 2) [29,30,31]. Additionally, the clade displays broad yet discrete thermal thresholds (~15–30 °C; Table 3 ), allowing clear empirical tracking of temperature-mediated range shifts. Finally, the robust genomic and transcriptomic resources curated for *Takifugu* allow researchers to dissect the precise molecular cascades linking ocean warming, hypoxia, and acidification to gene expression and transport physiology [17], highlighting both the utility of this model genus and the pressing need to validate these findings across non-model tetraodontid clades. 

## 4. Climate Drivers and Pufferfish Physiology

Global ocean change exposes pufferfishes to concurrent physical and chemical perturbations that act across multiple biological scales, impacting host physiology alongside the microbial and trophic networks governing TTX availability [36]. The following subsections evaluate the three principal climate drivers majorly elevated sea surface temperature, hypoxia, and ocean acidification by detailing their specific biochemical pathways, validated tetraodontid responses, and direct consequences for TTX allocation. Section 4.4 integrates these findings to address compound multi-stressor regimes, underscoring major gaps in current experimental frameworks. Although warming, hypoxia, and acidification act through distinct proximate mechanisms, the three subsections below converge on a shared physiological logic: each stressor forces a metabolic trade-off between cellular defence (antioxidant and heat-shock responses, ion regulation) and other energy-demanding processes, including reproductive investment and toxin translocation via ABC transporters. Read together, Section 4.1, Section 4.2 and Section 4.3 therefore describe not three independent pathways but one recurring allocation problem, energy diverted to stress defence versus energy available for TTX accumulation and provisioning, that manifests differently depending on which stressor and which life stage is involved. Section 4.4 then asks what happens when this allocation problem is triggered by more than one stressor simultaneously.

### 4.1. Ocean Warming: Thermal Stress Pathways and Links to TTX

Ocean warming affects pufferfish TTX dynamics through two distinct routes: (a) direct physiological effects on cellular integrity, metabolic allocation, and reproductive biology; and (b) indirect effects mediated by warming-driven shifts in TTX-producing bacterial communities and changes in the range and abundance of toxic prey species [1,37].

**Mechanistic pathway**

As poikilotherms, *Takifugu* species are directly susceptible to rising sea surface temperature, which drives proportional increases in metabolic rate and, above thermal optima (~22–24 °C for most species), progressive cellular damage [10,37]. At supra-optimal temperatures, overproduction of reactive oxygen species (ROS) overwhelms endogenous antioxidant defences, superoxide dismutase (SOD) and catalase (CAT), generating oxidative stress that elevates plasma biomarkers of tissue damage including aspartate aminotransferase (AST) and alanine aminotransferase (ALT) [27,28]. This thermal stress triggers a shift from adaptive success to the Intrinsic Pathway Cascade, forcing a metabolic trade-off where energy is prioritized for cellular defence (HSP/Antioxidants) over the maintenance of acquired TTX. Consequently, climate-driven thermal stress directly limits TTX accumulation and alters its allocation within the food web, increasing ecological risk, as illustrated in Figure 2 [29,31,38].

**Evidence in pufferfishes [M]**

Controlled experiments with *T. obscurus* at 34 °C versus 28 °C confirm temperature-dependent increases in DNA strand breaks (comet assay, haemocytes, and liver cells), activation of the p53–BAX apoptotic signalling pathway, and upregulation of HSP70 and HSP90 [24,27,28]. The energetic cost of sustained HSP expression creates documented trade-offs in growth and condition, relevant to reproductive output and, by extension, maternal toxin transfer to eggs [27,39].

**Link to TTX availability [SA] + [H]**

(a) Direct effects—toxin allocation: At the tissue level, warming-induced oxidative stress may alter the expression of ABC transporters involved in TTX translocation from the gut to liver and ovaries [18,20]. Field data consistently show that tissue TTX, particularly hepatic and ovarian TTX in *T. niphobles* and *T. pardalis*, peaks during warmer months coinciding with reproductive season and elevated sea surface temperature [29,40,41]. However, spawning seasonality, prey availability, and bacterial community shifts all co-vary with temperature, so causal attribution from field data alone is not possible. [H] We hypothesize that thermal stress sufficient to alter ABC transporter expression may modulate toxin compartmentalization efficiency into reproductive tissues, a prediction requiring isothermal TTX-feeding experiments spanning the thermal tolerance range.

(b) Indirect effects—microbial communities and prey availability: Warming co-drives seasonal proliferation of TTX-producing bacteria (*Vibrio* spp., *Aeromonas* spp.) in coastal waters, indirectly increasing dietary TTX availability [2,38,42]. Simultaneously, the poleward expansion of toxic prey species, including the flatworm *Planocera multitentaculata*, under warming conditions increases dietary TTX exposure in newly colonized regions [8,29].

**Early life stage vulnerability [M]**

Increasing incubation temperature from 19 °C to 28 °C in *T. obscurus* accelerated embryonic development but significantly reduced total hatch rates at 28 °C, defining a narrow thermal optimum for early survival [15,32]. These recruitment effects, if sustained under projected warming scenarios, could alter age-structure and population dynamics of pufferfish in warming coastal systems.

### 4.2. Hypoxia: Respiratory Adaptation and Potential Toxin Consequences

Like ocean warming, hypoxia influences TTX dynamics through two routes: (a) direct physiological effects on pufferfish metabolic allocation, energy budgets, and endocrine regulation of reproductive toxin provisioning; and (b) indirect effects mediated by hypoxia-driven restructuring of benthic and water-column microbial communities, which alters environmental TTX availability [3,35].

**Mechanistic pathway**

**Figure 3:**
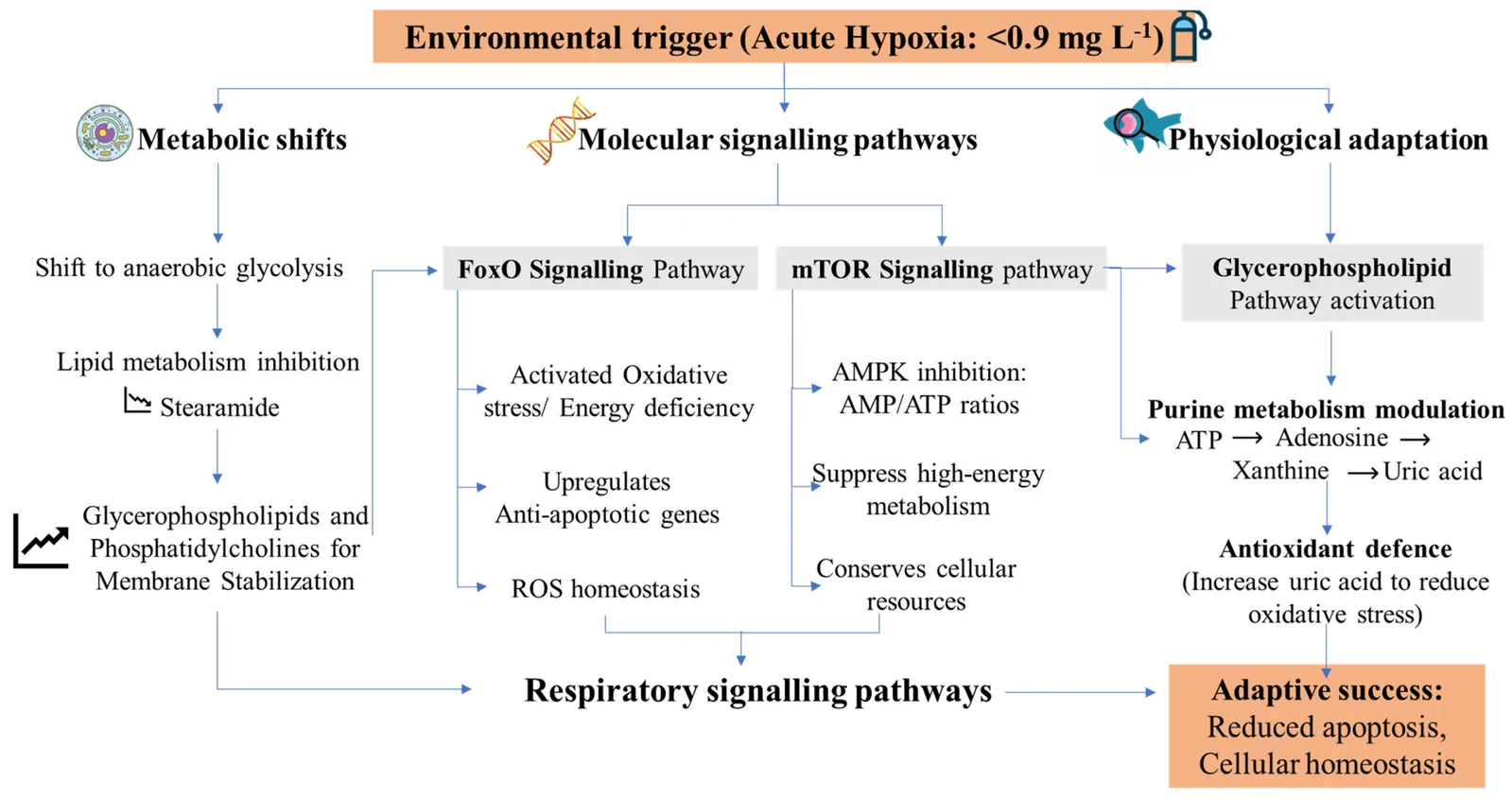
Hypoxia-induced molecular adaptation pathways in pufferfish [27]. Acute hypoxia (<0.9 mg L^−1^ dissolved oxygen) activates key signalling pathways including FoxO and mTOR, regulating stress responses and energy metabolism. Glycerophospholipid metabolism is enhanced to maintain membrane integrity under oxygen-limited conditions. These coordinated responses reduce apoptosis and support cellular homeostasis, enabling survival under hypoxic stress. The irreversible suppression of steroid-hormone metabolism under hypoxia may disrupt endocrine regulation of reproductive TTX allocation, and restructuring of benthic microbial communities toward hypoxia-tolerant taxa may alter environmental TTX availability, together representing an indirect but ecologically significant route through which coastal deoxygenation modifies pufferfish toxin dynamics [12,35].

Coastal hypoxia, driven by seasonal thermal stratification and eutrophication and exacerbated by warming, is increasing in frequency and geographic extent [11]. *Takifugu* species are susceptible to oxygen limitations owing to restricted gill surface area relative to body mass [3]. Critical oxygen tension (P_crit_), the dissolved oxygen (DO) concentration at which aerobic regulation gives way to oxy-conformation, has been measured at approximately 1.5–1.6 mg L^−1^ in juvenile *T. fasciatus* [3]. As shown in Figure 3, this response involves a shift to anaerobic glycolysis and inhibited lipid metabolism to facilitate membrane stabilization, alongside FoxO and mTOR signalling to regulate ROS homeostasis and suppress energy-intensive processes. Concurrently, purine metabolism increases uric acid production to mitigate oxidative stress, collectively ensuring cellular homeostasis [22].

These metabolic and molecular adjustments likely alter the energetic budget available for TTX accumulation and sequestration [43]. By prioritizing survival, hypoxic stress may disrupt metabolic homeostasis, potentially forcing a reallocation of toxins between skin and viscera as a defensive response [44]. Ultimately, these pathways act as functional regulators of toxin dynamics, suggesting that environmental hypoxia may fundamentally intensify the ecological risks and chemical ecology of *Takifugu* species.

**Evidence in pufferfishes [M]**

Under acute hypoxia (DO ≈ 0.9 mg L^−1^), HIF-1α is stabilized and translocates to the nucleus to activate genes encoding glucose transporters (GLUT2), glycolytic enzymes, erythropoietin (EPO), and vascular endothelial growth factor A (VEGF-A) [3,34,45]. Post-hypoxic hemoglobin and haematocrit levels increase substantially in *T. rubripes*, indicating enhanced oxygen-carrying capacity as an adaptive response [3]. Metabolomic profiling of *T. obscurus* gills under acute hypoxia reveals suppression of fatty-acid β-oxidation, upregulation of glycerophospholipid biosynthesis, irreversible reduction of steroid-hormone metabolites (including testosterone, persisting after reoxygenation), and activation of FoxO and mTOR signalling pathways governing apoptosis resistance and energy conservation [35].

**Link to TTX availability [H]**

No study has directly tested whether hypoxia modifies TTX accumulation in pufferfishes. Two plausible routes merit experimental investigation.

(a) Direct effects—reproductive toxin allocation: Irreversible hypoxia-induced suppression of steroid-hormone metabolism [35] could disrupt the endocrine regulation of reproductive toxin allocation, potentially decoupling the normal co-amplification of gonad maturation and ovarian TTX observed in normoxic conditions.

(b) Indirect effects—microbial restructuring: Restructuring of benthic and water-column microbial communities under hypoxia (toward anaerobic or hypoxia-tolerant taxa) may alter the relative abundance of TTX-producing bacteria in the near-field environment of pufferfishes, modifying dietary TTX availability [27].

### 4.3. Ocean Acidification: Sensory and Behavioural Disruption

Work in other teleosts underscores that acidification effects are frequently indirect and condition-dependent, acting through neuroendocrine stress-axis regulation (delayed post-stress cortisol recovery, altered neurotransmitter balance) rather than a single direct toxic pathway [46], a pattern consistent with the mixed effects reported for pufferfishes below.

Ocean acidification affects TTX dynamics through (a) direct effects on pufferfish neurophysiology, behaviour, and reproductive signalling, which may disrupt TTX-mediated chemical communication during spawning; and (b) indirect effects on microbial community composition and prey toxin bioavailability, though both routes remain at the hypothesis stage [12,47].

**Mechanistic pathway**

Ocean acidification, the progressive reduction in seawater pH driven by dissolution of anthropogenic CO_2_, causes respiratory acidosis in fishes when internal pH drops [47]. *Takifugu* and related pufferfishes compensate primarily by retaining bicarbonate (HCO_3_^−^) across the gills while excreting H^+^, maintaining extracellular pH homeostasis [18,24].

**Evidence in pufferfishes [M]**

Early life stages are significantly more vulnerable to ocean acidification than adults. In T. obscurus, reduced pH (pH 5.0) significantly delayed hatching relative to neutral or alkaline conditions, extreme acidification was lethal, and synergistic temperature × pH interactions on development were confirmed [15]. Larval viability at 24 h post-hatch declined sharply at pH 5.0 relative to pH 6.0–8.0, and abnormality rates increased markedly [15]. These thresholds are more extreme than open-ocean ocean acidification projections (current pH ≈ 8.1, projected ≈ 7.8–7.9 by 2100), but estuarine and coastal populations regularly experience more severe pH excursions.

At the organismal level, the most ecologically significant impact of ocean acidification may be disruption of neurosensory and behavioural function mediated via GABA receptors [18,48]. Intracellular accumulation of HCO_3_^−^ and reduction of Cl^−^ during acid–base compensation can reverse the electrochemical gradient across neuronal membranes, causing GABA receptors to become excitatory rather than inhibitory, leading to impaired predator avoidance and foraging efficiency.

**Link to TTX dynamics [H]**

(a) Direct effects—reproductive signalling: Since TTX may function as a pheromonal signal during spawning coordination in some pufferfish species [48], ocean acidification-driven disruption of olfactory sensitivity could impair TTX-mediated reproductive signalling, with downstream effects on spawning synchrony and maternal toxin transfer to eggs. This hypothesis requires targeted experimental testing and should not be overstated given current evidence.

(b) Indirect effects—microbial community: [H] Reduced pH may alter microbial community composition in ways that affect environmental TTX availability, but the direction and magnitude of this effect are unknown [12].

### 4.4. Multistressor Interactions: A Critical Knowledge Gap

While individual climate drivers have typically been evaluated in isolation, their interactive and compounding effects remain virtually uncharacterized within marine TTX dynamics. Embryonic exposure trials offer the sole direct evidence of stressor interactivity in tetraodontids: documented temperature × pH interactions on *T. obscurus* hatching kinetics [15] demonstrate that warming and acidification act synergistically on developmental endpoints in ways that cannot be extrapolated from isolated single-stressor exposure curves. This unpredictability reflects a broader paradigm in marine multi-stressor research; a comprehensive synthesis on coastal fisheries management confirmed that concurrent warming, deoxygenation, and acidification elicit complex additive, synergistic, or antagonistic responses that vary across ontogenetic stages and taxonomic lineages without uniform predictability [36]. Factorial exposure trials across non-teleost taxa similarly demonstrate that this oceanographic ‘deadly trio’ induces non-linear physiological and behavioural disruptions [39], establishing multi-stressor TTX investigations as an urgent research imperative rather than an exploratory elective.

We hypothesize that combined thermal–hypoxic stress, a combination increasingly common in stratified coastal systems, may divert metabolic resources from reproductive investment while simultaneously altering TTX allocation to gonads. The irreversible steroid-hormone disruption documented under hypoxia [35] could interact with warming-driven acceleration of reproductive timing, generating reproductive asynchrony that reduces toxin provisioning in eggs. These predictions have not been experimentally tested in any pufferfish species. Priority multistressor experiments should test pairwise combinations of warming (ambient +2 °C, +4 °C), hypoxia (DO reduced to 50% and 25% saturation), and acidification (ΔpH −0.3, −0.5 units) on TTX accumulation, tissue distribution, and analogue profiles over ecologically relevant exposure durations (8–12 weeks), using the LC-MS/MS framework described in Section 6. The following table summarizes quantified physiological and toxicological responses across pufferfish species and climate drivers discussed in Section 4 and Section 5 (Table 4).

## 5. Climate-Mediated TTX Dynamics: Microbial, Trophic, and Hybrid Pathways

Climate change influences TTX dynamics through interconnected microbial, trophic, and biogeographic pathways. Current empirical consensus establishes that tetrodotoxin (TTX) originates primarily from symbiotic and environmental bacterial consortia rather than endogenous eukaryotic biosynthesis, with genera including *Vibrio*, *Aeromonas*, *Pseudomonas*, *Shewanella*, and *Raoultella* identified as primary toxigenic candidates [4,8,38]. In vitro investigations demonstrate that TTX-producing strains, particularly *Vibrio* spp., exhibit accelerated proliferation kinetics and enhanced toxin biosynthesis at elevated thermal regimes (20–30 °C), while anthropogenic ocean warming continues to drive the geographic expansion and demographic proliferation of *Vibrio* assemblages across coastal waters [2,38]. Corroborating these microbiological patterns, in situ field surveys document elevated somatic and gonadal TTX titers in pufferfishes during warm seasons and peak spawning windows, although disentangling the confounding influences of temperature-stimulated uptake from endogenous reproductive cycles remains an analytical challenge [40,41,49]. Rising sea temperatures have also been associated with TTX occurrence in previously unaffected regions, including British coastal waters and potentially parts of sub-Saharan Africa, suggesting climate-driven geographic expansion of TTX-producing microorganisms [8,9]. However, substantial strain-level variation exists, and increased bacterial abundance does not necessarily translate directly into increased environmental TTX [4,38].

TTX accumulation in pufferfish occurs primarily through trophic transfer. Fish acquire toxin by consuming TTX-bearing prey such as toxic flatworms, gastropods, starfish, and sediment-associated organisms, while intestinal microbiota may further influence bioaccumulation processes [1,20]. Multi-omics approaches are beginning to resolve the molecular basis of this accumulation: transcriptomic and proteomic profiling in *T. bimaculatus* has identified differentially expressed genes and proteins in liver and ovarian tissue following TTX exposure, implicating hepatic detoxification and ovarian transport pathways in toxin handling [50], while comparable transcriptome–proteome work in *T. flavidus* skin points to cardiac-muscle contraction and adrenergic signalling genes as candidate accumulation pathways [51]. Ocean warming continues to drive the poleward displacement of toxic prey assemblages, establishing novel trophic pathways for TTX bioaccumulation in temperate and sub-polar regions [8,29]. Within host reproductive biology, maternal offloading of TTX into mature clutches provides essential early-life chemical protection against predation, reflected by marked seasonal elevations in ovarian toxin burdens synchronized with spawning [14,40,49]. Furthermore, temperature-induced upper-ocean stratification enhances aggregate marine snow formation and microbial dispersion, potentially amplifying vertical and horizontal TTX trophic flux [1]. While ocean acidification may concurrently alter toxin uptake and cellular binding dynamics across intermediate prey organisms, the magnitude and ecological significance of these carbonate chemistry effects remain largely uncharacterized [8,52]. Climate-driven range shifts are altering pufferfish distributions worldwide. In the Northwest Pacific, increasing sea-surface temperatures have promoted the northward expansion of *Takifugu* species into regions such as Tohoku and Hokkaido [8,34]. Similarly, the invasive pufferfish *L. sceleratus* has rapidly expanded throughout the Mediterranean since its first record in Turkey in 2003, with warming conditions enhancing establishment success and increasing human poisoning risks due to high tissue TTX concentrations [13,25]. Climate change may also favour habitat-generalist and invasive pufferfish species while reducing habitat suitability for more specialized taxa, thereby restructuring fish communities and toxin pathways [22].

A critical emerging concern involves climate-facilitated hybridization among previously allopatric *Takifugu* species. As climate-driven range shifts expand zones of sympatry, introgressive hybridization frequencies increase, yielding hybrid cohorts with non-canonical and highly erratic TTX anatomical distributions [13,14,17]. In contrast to parental lineages, whose somatic muscle tissue is conventionally non-toxic and commercially harvested, hybrid individuals can sequester hazardous TTX titers within edible musculature—a phenomenon termed ‘cryptic toxicity’ that is morphologically indistinguishable and mandates analytical chemical surveillance [14,20]. Proposed molecular drivers include the disruption or novel recombination of active toxin transporters, host-associated microbiome remodelling, and divergent expression of soluble TTX-binding proteins, though these candidate mechanisms remain theoretical hypotheses [H] requiring targeted empirical testing [1,20].

TTX occurs alongside several structurally related analogues, including 4,9-anhydro-TTX, 11-oxo-TTX, 6-epi-TTX, and 11-deoxyTTX, which differ in toxicity and tissue distribution [20,31,53]. Dietary studies confirm that both TTX and its analogues can be accumulated through feeding [54]. Recent evidence suggests that analogue composition varies seasonally and may be influenced by temperature, with warmer conditions associated with higher proportions of 4,9-anhydro-TTX [41]. Because traditional mouse bioassays cannot distinguish among analogues, they may underestimate overall neurotoxic risk. In contrast, LC-MS/MS enables simultaneous quantification of individual analogues, and reporting toxicity as TTX-equivalents is increasingly recommended for accurate risk assessment [26,53,54]. 

## 6. Analytical Methods and Evidence Assessment

Methodological advancements in analytical instrumentation have markedly enhanced the sensitivity, resolution, and structural characterization of tetrodotoxin (TTX) and its associated analogues across complex biological and environmental matrices. The traditional mouse bioassay, although still used for regulatory purposes in Japan, has limited sensitivity (~2 µg MU g^−1^ tissue), cannot distinguish among TTX analogues, and raises ethical concerns, making it unsuitable for detecting subtle climate-driven changes in toxin dynamics [53]. In contrast, LC-MS/MS and UHPLC-MS/MS provide highly sensitive and accurate quantification (0.05–0.5 µg g^−1^ tissue), allowing simultaneous detection of multiple analogues and enabling the assessment of seasonal and spatial variation in TTX profiles associated with environmental change [9,10,53,55]. This section evaluates the major approaches in terms of their suitability for climate-linked TTX research (summarized in Table 5), and concludes with a structured assessment of the overall evidence base (Table 6).

Organ-specific analysis is vital given the marked compartmentalization of TTX in teleost hosts, where hepatic, cutaneous, and ovarian tissues exhibit selective bioaccumulation, in contrast to the generally benign musculature of purebred taxa [40,49]. Profiling multiple anatomical matrices is thus imperative to capture climate-mediated alterations in tissue-partitioning kinetics and screen for aberrantly toxic hybrid lineages. At the microbiome interface, next-generation sequencing including 16S rRNA profiling and functional metagenomics elucidates taxonomic composition and candidate biosynthetic pathways of TTX-producing bacterial consortia; however, because genomic presence does not demonstrate active secondary metabolite synthesis, these molecular tools require coupled analytical verification [1]. Furthermore, stable isotope ecology represents a powerful tool to delineate dietary TTX bioaccumulation pathways and assess trophic disruptions under climate stress, though its broader adoption remains limited by procedural complexity and cost [1]. A major challenge for advancing climate–TTX research is the lack of internationally standardized analytical protocols. Differences in detection methods, reporting units, tissue selection, and access to analytical infrastructure hinder comparisons among regions and limit the feasibility of global meta-analyses. Greater adoption of LC-MS/MS-based methods and harmonized reporting standards would substantially improve the reliability and comparability of future climate–TTX datasets [56]. Beyond targeted chemical assays, multi-omics profiling (transcriptomics, proteomics) is emerging as a complementary tool for climate–TTX research. Rather than only measuring toxin concentration, these approaches identify the specific genes, proteins, and pathways (e.g., ABC-transporter and detoxification machinery) that govern toxin uptake, transport, and tissue-specific accumulation [50,51,57], and could in principle be applied to test whether warming- or hypoxia-induced gene-expression shifts (Section 4 and Section 5) causally alter toxin allocation, a link that is currently only hypothesized [H] in this review.

The following table summarizes climate-sensitivity tolerance thresholds across pufferfish species and life stages discussed in Section 4 (Table 6).

## 7. Public Health Implications

Climate change reshapes pufferfish TTX risk in at least three ways with direct public health relevance. First, warming expands the geographic range of TTX-bearing species into regions without monitoring infrastructure or regulatory experience, as exemplified by *L. sceleratus* in the Mediterranean [13,25,58] and the northward extension of *Takifugu* species in Japan. Second, cryptic hybrid toxicity in newly sympatric zones produces tissue-TTX patterns that violate species-based food-safety assumptions [14,59]. Third, climate-associated changes in bacterial community dynamics may alter the timing and magnitude of seasonal TTX peaks, rendering historical safety calendars less reliable [33,41,42].

Contemporary management frameworks remain hindered by technical and operational deficiencies: obsolete mouse bioassay protocols lacking congener resolution, diagnostic reliance on external morphology in the face of widespread hybridization, static regulatory calendars outpaced by climate-induced seasonal shifts, and poor interoperability among national surveillance programmes. A unified ‘One Health’ framework offers a cohesive, cross-sectoral solution to this systemic vulnerability [1,52]. By systematically coupling in situ environmental surveillance (temperature, hypoxia, acidification, and microbial proliferation) [12,60,61] with rigorous food-safety workflows (tissue-level TTX and congener quantification alongside genetic verification) [16,62,63] and clinical epidemiological intelligence (rapid poisoning reporting systems) [38,64,65], this strategy establishes a synchronized defence across ecological habitats, commercial supply chains, and consumer populations. Specific monitoring recommendations:

1. Deploy seasonal LC-MS/MS-based TTX surveillance in high-risk commercial species, covering multiple tissues (liver, skin, ovary, muscle) with sampling intervals of ≤2 months during the warm season;
2. Integrate sea surface temperature, DO, and pH monitoring data with toxin surveillance records to build predictive correlation models for high-risk seasons;
3. Implement molecular identification (DNA barcoding or species-specific PCR) as a routine first step for commercial pufferfishes caught in regions experiencing range expansion or hybridization;
4. Establish analogue profiling (TTX + 4,9-anhydro-TTX + other analogues) as standard practice, replacing total-toxicity bioassays, to enable risk assessment based on toxicity equivalents;
5. Evaluate plant-based antiparasitic strategies for farmed pufferfishes as a low-impact complement to conventional disease management under warming conditions [50];
6. Apply multi-omics (transcriptomic/proteomic) profiling across warming, hypoxia, and acidification treatments to directly test whether climate stressors alter expression of TTX transport and detoxification genes, moving current allocation hypotheses [H] toward confirmed mechanism [M] status.

## 8. Conclusions

Pufferfishes offer an exceptionally tractable model system for investigating climate-mediated shifts in marine toxin landscapes. Because tetrodotoxin (TTX) is acquired exogenously through bacterial biosynthesis and trophic bioaccumulation rather than autogenous production, any environmental perturbation that reshapes microbial physiology, food-web architecture, or biogeographic ranges intrinsically modulates host toxicity dynamics. This systematic scoping review synthesizes empirical and theoretical evidence across 62 primary studies to delineate three principal mechanistic pathways linking anthropogenic climate drivers to tetraodontid TTX dynamics.

**Key Findings**

**1. Microbial** and trophic pathways are climate-sensitive [M + SA]. Laboratory evidence confirms that Vibrio and related TTX-producing bacteria show temperature-dependent increases in growth rate and toxin production (confirmed mechanism [M]). Field observations from Japan and Korea consistently show higher tissue TTX in warm months, coinciding with bacterial proliferation and spawning season (strong association [SA]); however, the relative contributions of bacterial, dietary, and reproductive mechanisms cannot be partitioned from field data alone.

**2. Range shifts and hybridization create novel toxicity landscapes [SA].** Climate-driven northward expansion of *Takifugu* species in the Northwest Pacific and the Lessepsian establishment of *Lagocephalus sceleratus* in the Mediterranean are well-documented [SA]. Species distribution models projecting *L. sceleratus* habitat suitability under realistic warming scenarios indicate continued expansion into the Adriatic, Ligurian, and western Mediterranean basins, with maximum seawater temperature as the strongest predictor of establishment [66,67], reinforcing food-safety concern given TTX detections already reported across Mediterranean seafood [68].

In both contexts, novel sympatry among previously isolated species increases hybridization frequency. Hybrid individuals exhibit TTX in muscle, tissue considered safe in parental species, constituting a cryptic food-safety hazard not detectable by morphological species identification or conventional bioassay.

**3. Physiological stress responses are established; their links to TTX allocation remain hypothetical [M + H].** Warming, hypoxia, and acidification each produce well-characterized physiological responses in *Takifugu* [M]. These responses plausibly alter toxin allocation and accumulation through multiple mechanisms, but no controlled experiment has directly tested multistressor effects on TTX dynamics [H].

**Critical Knowledge Gaps**

(1) Long-term (≥5 years) field monitoring integrating environmental variables and tissue TTX across multiple regions is absent;
(2) The molecular basis of hybrid cryptic toxicity is completely unknown;
(3) No controlled experiment has tested multistressor effects (combined warming + hypoxia ± acidification) on TTX accumulation, analogue profiles, or tissue distribution;
(4) TTX analogue climate sensitivity is unexplored; preliminary field data suggest analogue ratios may be temperature-dependent [SA], but this requires replication [H];
(5) Tropical, Southeast Asian, and Indian Ocean pufferfish populations remain essentially unmonitored for climate-TTX dynamics.

**Recommendations**

(1) Adopt LC-MS/MS with full analogue profiling as the international standard for TTX monitoring, replacing the mouse bioassay.
(2) Implement DNA barcoding-based hybrid detection in commercial catches from regions experiencing range expansion.
(3) Prioritize multi-stressor TTX-accumulation experiments (warming × hypoxia, 8–12 weeks) as the highest-value near-term research investment.
(4) Develop integrated environmental–toxin surveillance networks linking oceanographic monitoring to seafood safety programmes, enabling proactive early-warning of high-risk periods.
(5) Prioritize metagenomic surveys-including 16S rRNA amplicon sequencing and functional metagenomics targeting TTX biosynthesis gene clusters of TTX-associated bacterial communities across seasonal and thermal gradients, paired with simultaneous LC-MS/MS chemical quantification of tissue and environmental TTX. This pairing is essential to establish whether, and by how much, climate-driven shifts in microbial community composition translate into changes in environmental TTX availability and pufferfish tissue burden.
(6) Develop regional risk maps integrating sea surface temperature, dissolved oxygen concentration, pufferfish species distribution records, and historical TTX poisoning incident data. Such maps would identify priority surveillance zones, particularly in Southeast Asia, the Indian Ocean, and newly colonized Mediterranean populations, and enable proactive, evidence-based management responses in regions currently lacking monitoring infrastructure.

Understanding pufferfish toxification as a dynamic, climate-sensitive ecological process rather than a fixed species property is the prerequisite for anticipating where and when the next emergence of TTX in a new region or species will occur, and for building the monitoring and regulatory capacity to respond before poisoning incidents result in preventable harm.

## Figures and Tables

**Table 1:** Characteristics of included studies (*n* = 62).

Category	Characteristic	Studies (n)	%
Publication trend	Published 2015–2024	39	63
Geographic distribution	East Asia (Japan *n* = 22)	31	50
Geographic distribution	Southeast Asia	15	24
Geographic distribution	Mediterranean/Atlantic	8	13
Geographic distribution	Oceania	5	8
Study design	Field observational	34	55
Study design	Laboratory experiments	18	29
Study design	With quantitative TTX data	28	45
Climate variable addressed	Temperature/warming	48	77
Climate variable addressed	Oxygen/hypoxia	18	29
Climate variable addressed	Salinity/estuarine	12	19
Climate variable addressed	Ocean acidification (pH)	8	13
Climate variable addressed	Multi-stressor design	11	18
Study metrics (median, range)	Sample size (individuals)	85	12–2400
Study metrics (median, range)	Study duration (years)	2.5	<1 month to 12 year
Study metrics (median, range)	Sampling sites/populations	3	1–18
Analytical method used	TTX quantitative reporting	42	68
Analytical method used	HPLC or LC-MS analysis	31	50

**Table 2:** Harvest Plot: Climate-TTX associations (*n* = 28 Studies).

Climate Variable	Increased TTX	Decreased TTX	No Change	Total	Consistency
Temperature	14 (58%)	3 (13%)	7 (29%)	24	Mixed
Hypoxia	8 (73%)	1 (9%)	2 (18%)	11	Consistent
pH/Acidification	2 (40%)	1 (20%)	2 (40%)	5	Conflicting
Salinity	4 (57%)	1 (14%)	2 (29%)	7	Mixed
Multi-stressor	4 synergistic	-	2 additive	6	Synergistic

**Table 3:** Comparative climate sensitivity across pufferfish species and life stages. Tolerance limits derived from experimental studies.

Species (Common Name)	Life Stage	Stressor	Reported Tolerance/Response	Reference
*T. fasciatus* (*Striped puffer*)	Immature juvenile	Dissolved oxygen	Acute hypoxia tolerated at ~1.6 mg L^−1^, distress below ~1.5–1.6 mg L^−1^, normoxia ~7 mg L^−1^	[3]
*T. flavidus* (*Tawny puffer*)	Larvae	Temperature	Optimal range 23–29 °C, survival declines outside this range	[32]
*T. flavidus*	Egg	Temperature	Optimal incubation range 23–26 °C	[32,33]
*T. obscurus* (*Obscure puffer*)	Embryo	pH	Development impaired at extremes, near-normal at pH 6.5–9.0, lethal at pH 5.0	[15]
*T. obscurus*	Larva	Temperature	Optimal survival 22–23 °C, significant deformities at 19 °C, viability declines > 25 °C	[15,31]
*T. obscurus*	Embryo	Temperature × pH	Significant synergistic interaction: acidification increasingly delays development at lower temperatures	[15]
*T. obscurus*	Embryo	Temperature (upper)	Total hatch rate significantly reduced at 28 °C compared to lower temperatures	[15]
*T. rubripes* (*Tiger puffer*)	Juvenile/Adult	Hypoxia	HIF-1α stabilization, increased haematocrit, hemoglobin post-hypoxia, EPO/VEGF-A upregulation	[3,34]
*T. obscurus*	Adult (gill tissue)	Hypoxia (<0.9 mg L^−1^ DO)	Metabolomic disruption: suppressed β-oxidation, irreversible steroid-hormone reduction, FoxO/mTOR activation	[35]

**Table 4:** Climate change-related environmental stressors and quantified physiological responses in pufferfishes. Evidence type: Experimental = controlled laboratory conditions, Field = observational field study.

Climate Driver	Species	Parameter Affected	Response Level	Quantified Finding	Evidence Type	Reference
Ocean warming (supra-optimal, 34 °C vs. 28 °C)	*T.* *obscurus*	Oxidative stress (SOD, CAT, plasma AST, ALT)	Cellular (molecular)	Significant elevation of oxidative stress markers, temperature-dependent DNA strand breaks in haemocytes	Experimental	[27,28]
Ocean warming (8–12 week exposure)	*T.* *obscurus*	p53–BAX apoptotic pathway, HSP70/90 expression	Cellular (molecular)	Apoptosis induction confirmed, HSP upregulation documented, energetic cost reduces growth	Experimental	[27,28]
Ocean warming (28 °C)	*T. obscurus* (*embryo*)	Time to 50% hatch	Physiological (developmental)	Accelerated: ~250 h (19 °C) → ~91 h (28 °C), total hatch rate significantly reduced at 28 °C	Experimental	[15]
Ocean warming + salinity shift	*T. fasciatus*	Apoptosis/antioxidant enzymes/MAPK pathway	Cellular (molecular)	Interactive effects documented, temperature × salinity interaction on apoptosis rate	Experimental	[39]
Hypoxia (<0.9 mg L^−1^ DO)	*T.* *obscurus*	Gill metabolome, lipid, amino acid, steroid metabolism	Cellular (metabolomic)	Fatty acid β-oxidation suppressed, testosterone reduced irreversibly, glycerophospholipid upregulation for membrane protection	Experimental	[35]
Hypoxia (acute, critical DO ~1.5–1.6 mg L^−1^)	*T. fasciatus*	Critical oxygen tension (P_crit_)	Physiological (whole organism)	Behavioural distress below ~1.5–1.6 mg L^−1^, normoxia ~7 mg L^−1^	Experimental	[3]
Hypoxia (HIF-1α pathway)	*T. rubripes*/*T. fasciatus*	Erythropoiesis, anaerobic energy pathways	Cellular/Physiological	HIF-1α stabilization, EPO/VEGF-A upregulation, increased hemoglobin/haematocrit post-hypoxia	Experimental	[3,34]
Ocean acidification (pH 5.0)	*T. obscurus* (*embryo*)	Hatch rate, larval viability, deformity rate	Physiological (developmental)	Hatch rate and viability significantly reduced at pH 5.0, deformity rate near zero at pH 6–8	Experimental	[15]
Temperature × pH interaction	*T. obscurus* (*embryo*)	Incubation duration	Physiological (developmental)	Acidification increasingly delays development at lower temperatures; synergistic interaction confirmed	Experimental	[15]
Ocean warming (seasonal, sea surface temperature 24–26 °C)	*T. niphobles*	Whole-body TTX content	Physiological (toxicological)	Significantly elevated during maturation/spawning season (warmer months) vs. “ordinary period”	Field (observational)	[40]
Ocean warming (seasonal)	*T.* *pardalis*	Hepatic TTX, gonadal TTX (ovary/testis)	Physiological (toxicological	Positive temperature–TTX correlation, peak during warm months, muscle generally non-toxic in wild-type	Field (observational)	[41]
Climate-driven warming (sea surface temperature)	Bivalves (UK coastal)	Tissue TTX accumulation	Physiological (toxicological	Sea temperature significantly predicts TTX in bivalves, higher TTX in warmer months	Field (observational)	[9]
Climate-linked Vibrio proliferation	*T. pardalis* (via food web)	Dietary TTX availability	Ecological (trophic)	Warmer sea surface temperature enhances TTX-producing Vibrio abundance, increased bioaccumulation inferred	Field + Review	[2,8]
Thermal stress → reduced cold extremes	*Tropical pufferfish* spp.	Overwintering survival in temperate latitudes	Population (distributional)	Improved temperate winter survival, population establishment in new regions documented	Field (distributional)	[22]
Climate-linked habitat degradation (coral bleaching)	*Canthigaster* spp., *Chelonodon pleurospilus*	Habitat availability/refugia loss	Population (ecological)	Warming-induced bleaching reduces live coral cover and seagrass, diminishing refugia and feeding grounds	Field	[22]

**Table 5:** Comparison of analytical methods for TTX detection and quantification in the context of climate-linked monitoring.

Method	Specificity	Quantitative?	Detects Analogues?	Detection Limit	Best Application
Mouse bioassay	Low, cannot distinguish TTX from analogues	Semi-quantitative (MU/g)	No	~2 µg MU/g	Historical datasets only; regulatory standard in Japan
LC-MS/MS	High-MS/MS fragmentation identifies each compound	Yes (µg/g ww)	Yes, resolves all major analogues simultaneously	0.05–0.5 µg/g	Risk assessment, climate monitoring, regulatory enforcement
UHPLC-MS/MS	High-as LC-MS/MS + better chromatographic resolution	Yes (µg/g ww)	Yes	0.05–0.5 µg/g	High-throughput environmental monitoring programmes
16S rRNA metagenomics	Medium-community composition (taxonomic)	Relative abundance only	No	N/A (gene-based)	Identifying TTX-producing bacteria, climate-community correlations
Functional metagenomics	Medium-detects biosynthesis gene clusters	Qualitative (gene presence)	No	N/A	Detecting TTX biosynthesis potential in environmental samples
Stable isotope analysis (13C, 15N, 34S)	Medium-trophic level and dietary source	Yes (isotope ratios)	No	N/A	Tracing trophic pathway of TTX from bacteria through food web to fish

**Table 6:** Evidence assessment and climate–TTX associations. Tier: [M]—confirmed mechanism, [SA]—strong association, [H]—hypothesis.

Key Claim/Climate Driver	Species/System	Evidence Type	n	Tier	References	Main Uncertainty/Gap
Warming increases growth and TTX production of *Vibrio* spp. in culture	*Vibrio* spp. isolates	Experimental (culture kinetics); confirmed higher growth/toxin production at 25–30 °C vs. 15–20 °C in axenic culture	~5	[M]	[2,38]	Strain-level variation: not all strains produce TTX; field-culture gaps remain
Climate-linked *Vibrio*/*Aeromonas* proliferation correlates with dietary TTX availability	*T. pardalis* (via food web)	Field inference; warmer SST correlated with higher Vibrio abundance, inferred increased dietary TTX → higher tissue burdens	-	[SA]	[8,9]	Correlative: dietary TTX increase inferred, not directly measured
Seasonal TTX peaks correlate with warm months (SST)	*T. niphobles*, *T. pardalis* (Japan, Korea)	Field observational; higher whole-body/hepatic/ovarian/testicular TTX in warm maturation/spawning months; analogue ratio (4,9-anhydro-TTX/TTX) higher in warm season	~8	[SA]	[40,41]	Spawning season, prey availability, and SST co-vary; cannot separate drivers from field data
Sea surface temperature predicts TTX in non-pufferfish taxa	Marine bivalves (UK)	Field (UHPLC-MS/MS); SST significantly predicts tissue TTX	-	[SA]	[9]	Demonstrates climate sensitivity outside pufferfish, but a single system
*L. sceleratus* establishment in Mediterranean linked to SST increase	*Lagocephalus sceleratus* (Mediterranean)	Distributional data + SDMs; tissue/muscle TTX frequently exceeds regulatory thresholds, poisoning incidents documented	~6	[SA]	[13,25]	Suez Canal widening and fishing pressure are co-occurring changes, climate attribution partial
Hybrid *Takifugu* exhibit TTX in muscle (cryptic toxicity)	*Takifugu* hybrids (Japan, Korea)	Field observational; significant muscle TTX detected in hybrids despite muscle being non-toxic in parental species	~3	[SA]	[14,20]	Small sample sizes; molecular basis of altered tissue distribution completely unknown
Bacterial TTX via novel host–parasite route	*Pseudocaligus fugu* (on *T. pardalis*)	Confirmed TTX accumulation in copepod; TTX-producing bacteria isolated directly from parasite	-	[M]	[5,6,7]	Route confirmed in one host–parasite system; generality untested
Warming-driven prey range shifts increase dietary TTX delivery	*Takifugu* spp. (via diet: flatworms, gastropods)	Field + trophic inference; northward expansion of toxic flatworms (*Planocera multitentaculata*) and gastropods increases dietary TTX delivery	~3	[SA]	[8,29]	Direct measurement of dietary TTX change under warming absent; trophic data correlative
Multi-stressor conditions (warming + hypoxia) alter pufferfish TTX allocation	*Takifugu* spp. (hypothesis)	Theoretical; hypothesized alteration of toxin allocation to reproductive tissues via steroid-hormone disruption	0 direct studies	[H]	[35]	Requires controlled 8–12 week multi-stressor TTX-feeding experiments

## Data Availability

The data supporting the findings of this review are available from the author upon reasonable request.
